# Evaluation of an Antibiotic Cocktail for Fecal Microbiota Transplantation in Mouse

**DOI:** 10.3389/fnut.2022.918098

**Published:** 2022-06-03

**Authors:** Jijun Tan, Jiatai Gong, Fengcheng Liu, Baizhen Li, Zhanfeng Li, Jiaming You, Jianhua He, Shusong Wu

**Affiliations:** Hunan Collaborative Innovation Center for Utilization of Botanical Functional Ingredients, College of Animal Science and Technology, Hunan Agricultural University, Changsha, China

**Keywords:** antibiotic cocktail, pretreatment, gut microbiota, mouse model, fecal microbiota transplantation

## Abstract

**Objective:**

This study aimed to evaluate the effect of an antibiotic cocktail on gut microbiota and provide a reference for establishing an available mouse model for fecal microbiota transplantation (FMT) of specific microbes.

**Design:**

C57BL/6J mice (*n* = 24) had free access to an antibiotic cocktail containing vancomycin (0.5 g/L), ampicillin (1 g/L), neomycin (1 g/L), and metronidazole (1 g/L) in drinking water for 3 weeks. Fecal microbiota was characterized by 16S rDNA gene sequencing at the beginning, 1st week, and 3rd week, respectively. The mice were then treated with fecal microbiota from normal mice for 1 week to verify the efficiency of FMT.

**Results:**

The diversity of microbiota including chao1, observed species, phylogenetic diversity (PD) whole tree, and Shannon index were decreased significantly (*P* < 0.05) after being treated with the antibiotic cocktail for 1 or 3 weeks. The relative abundance of *Bacteroidetes*, *Actinobacteria*, and *Verrucomicrobia* was decreased by 99.94, 92.09, and 100%, respectively, while *Firmicutes* dominated the microbiota at the phylum level after 3 weeks of treatment. Meanwhile, *Lactococcus*, a genus belonging to the phylum of *Firmicutes* dominated the microbiota at the genus level with a relative abundance of 80.63%. Further FMT experiment indicated that the fecal microbiota from the receptor mice had a similar composition to the donor mice after 1 week.

**Conclusion:**

The antibiotic cocktail containing vancomycin, ampicillin, neomycin, and metronidazole eliminates microbes belonging to *Bacteroidetes*, *Actinobacteria*, and *Verrucomicrobia*, which can be recovered by FMT in mice.

## Introduction

Fecal microbiota transplantation (FMT) has been widely used as an intervention method in the reconstruction of receptor gut microbiota in mice-model experiments against various diseases, such as obesity (1), colitis (2), *clostridium difficile* infection (3) and spinal cord injury (4). However, donor-derived microbiota transplanted into receptor guts is not easy to achieve colonization since indigenous microbiota is dominant and stable (5). Hence, suitable intestinal preparation is vital in the pretreatment of FMT to attain a susceptible microbiota environment. Antibiotic treatments for mice are more fundamental and economical than for germ-free (GF) mice because GF mice may induce global developmental changes in receptor guts, which can mask disease-specific attributes of donor material (6). However, methods of antibiotic treatments before FMT have been formed diversely, such as composition of antibiotics (7), administration time (8), and concentrations (9).

The antibiotic cocktail containing vancomycin, ampicillin, neomycin, and metronidazole have been adopted widely in mice (10–12). Vancomycin belonging to glycopeptide antibiotics, ampicillin belonging to beta-lactam antibiotics, neomycin belonging to aminoglycoside antibiotics, and metronidazole belonging to nitroimidazoles antibiotics constitute the most common classes of antibiotics against gram-positive bacteria, aerobes or anaerobes (13). The underlying antibacterial mechanisms of these single categories of antibiotics have been well studied (14, 15). However, studies that aim to assess the influence of antibiotic cocktails on different specific microbes are rare, although the antibiotic cocktail may lead to loss of diversity and changes in microbes’ composition (16, 17). Importantly, pieces of evidence are needed to understand the characteristics of microbes after treatment with the antibiotic cocktail to ensure the availability of the antibiotic cocktail and establish an FMT-based non-specific microbe mouse model. Consequently, this study aimed to evaluate the effect of an antibiotic cocktail containing vancomycin, ampicillin, neomycin, and metronidazole in drinking water on the composition of gut microbiota, and further verify the effectiveness of FMT.

## Materials and Methods

### Diets and Reagents

Mouse feed (D12450J) was purchased from Research Diets Inc. (New Brunswick, NJ, United States), and the diet composition was shown in Supplementary Table 1. Antibiotics including vancomycin, ampicillin, neomycin, and metronidazole were purchased from Shanghai Yuanye Bio-Technology Co., Ltd. (Shanghai, China).

### Mouse Model and Experimental Design

The experimental procedures were approved by the Hunan Agricultural University Institutional Animal Care and Use Committee (Permission No. 2020A34). Mice and sterilized poplar bedding were purchased from Hunan Slake Jingda Laboratory Animal Co., Ltd. (License No. SCXK-Xiang 2019-0004, Changsha, Hunan, China). Mice were housed separately in cages with a sterile environment under controlled temperature (23.5°C) and light (12 h light/day) and had free access to feed, drinking water, or an antibiotic cocktail containing vancomycin (0.5 g/L), ampicillin (1 g/L), neomycin (1 g/L), and metronidazole in drinking water.

**FIGURE 1:**
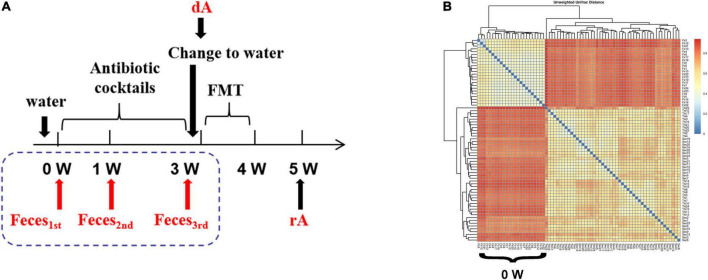
The cluster of fecal microbiota. **(A)** Experimental design drawing. Receptor mice had free access to antibiotic cocktails containing vancomycin (0.5 g/L), ampicillin (1 g/L), neomycin (1 g/L), and metronidazole (1 g/L) in drinking water. Feces were collected on the first day of the experiment (0 W), the first week of the experiment (1 W), the third week of the experiment (3W), as well as feces from donor mice (dM) fed with normal diet and feces from receptor mice (rM) when FMT ended over another 1 week. All mice were fed a normal diet. **(B)** β-diversity indices of unweighted_unifrac_distance. The redder color is, the farther distance is. dM, donor mice fed with a normal diet; FMT, fecal microbiota transplantation; PLS-DA, partial least squares discriminant analysis; rM, receptor mice transplanted with microbiota from dM.

A total of twenty-four C57BL/6J mice (SPF class, male, and 4 weeks of age) were raised separately with free access to an antibiotic cocktail containing vancomycin (0.5 g/L), ampicillin (1 g/L), neomycin (1 g/L), and metronidazole (1 g/L) in drinking water for consecutive 3 weeks. Then, mice were treated with clean drinking water for 3 days to avoid the interference of antibiotic cocktails on FMT, followed by FMT for 1 week. FMT experiment was conducted with a modified method based on Gong et al. (12). Briefly, fecal samples were collected sterilely through mild stimulation of the anus in mice, and resuspended in sterile phosphate buffer solution (PBS) at 0.125 g/ml, followed by low-speed (800 × *g*) centrifugation (Low-Speed Bench Centrifuge, TD4 angle rotor, Hunan Michael Laboratory Instrument Co., Ltd., Changsha, China) for 10 min. Ultimately, 0.15 ml of that supernatant was administered to mice by oral gavage once a day for up to consecutive 7 days. Feces of mice at the beginning of the experiment (0 W), the first week (1 W), the third week (3 W), as well as feces from donor mice (dM) and receptor mice (rM) were collected for characterization of microbiota (Figure 1A).

### Analysis of Gut Microbiota

Total DNA of feces was extracted by using a Stool DNA Isolation Kit (Tiangen Biotech Co., Ltd., Beijing, China). DNA quality was detected and controlled by Nanodrop (Thermo Fisher Scientific Inc., Rockford, IL, United States), and then 30 ng DNA was used for PCR amplification. The V4 hypervariable region of the bacterial 16S rRNA gene was amplified by PCR, where the forward primer was 515a: 5′–GTGCCAGCMGCCGCGGTAA-3′ and the reverse primer was 806: 5′-GGACTACHVGGGTWTCTAAT-3′. For each sample, a 10-digit barcode sequence was added to the 5′ end of the forward and reverse primers (provided by Allwegene Technology Inc., Beijing, China), and each sample was carried out by three same replications to mitigate reaction-level PCR biases. The volume of PCR reaction was 25 μL, containing 12.5 μL of 2xTaq PCR MasterMix, 1 μL of forward and 1 μL of reverse primers at the concentration of 5 μM, respectively, 3 μL BSA at the concentration of 2 ng/μL, 3 μL DNA samples (30 ng), and 4.5 μL double-distilled H_2_O (ddH_2_O). Cycling parameters were 95°C for 5 min, followed by 25 cycles at 95°C for 45 s, 50°C for 50 s, and 72°C for 45 s, and a final extension at 72°C for 10 min. Detection parameters of agarose gel electrophoresis were referred to as follows: concentration of gel with 1%, voltage with 170V, and electrophoretic time of 30 min. Three PCR products from the same sample were mixed in equidensity ratios and purified with a GeneJET Gel Extraction Kit (Thermo Fisher Scientific Inc., Rockford, IL, United States), quantified using real-time PCR, and sequenced at Allwegene Technology Inc. (Beijing, China).

### Statistical Analysis

Results are expressed as means ± *SD*. Significant differences between groups were determined using one-way ANOVA tests, followed by Fisher’s least significant difference (LSD) and Duncan’s Multiple Range test (SPSS21, IBM Corp., Armonk, NY, United States). A probability of *P* < 0.05 was considered significant.

## Results

### The Effect of Antibiotic Cocktail on a Cluster of Fecal Microbiota

As shown in Figure 1B, treatment with the antibiotic cocktail for 1 or 3 weeks induced obvious changes in microbiota clusters, and the evolutionary tree between 1 and 3 weeks suggested a similar sample composition.

### The Effect of Antibiotic Cocktail on α Diversity of Microbiota

**FIGURE 2:**
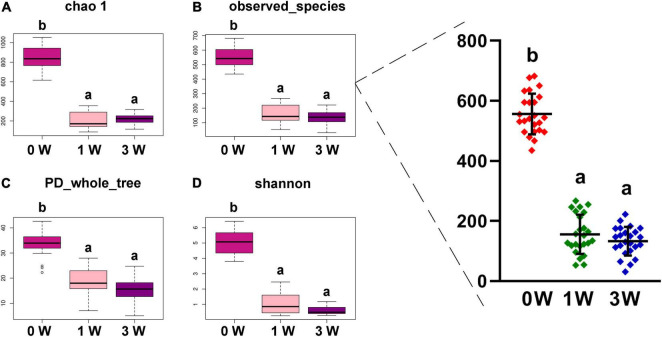
The α diversity of fecal microbiota. **(A)** Chao 1. **(B)** Observed species. **(C)** PD whole tree. **(D)** Shannon. Data represented as mean ± SD (*n* = 24). Bars with different letters differ significantly (*P* < 0.05). Chao1, observed species, and PD whole tree are species richness indices, and the Shannon index reflects the diversity of gut microbiota. PD, phylogenetic diversity.

The abundance of gut microbiota is associated with the stability of the micro-ecological environment. As shown in Figure 2, the antibiotic cocktail led to a significant decrease in α diversity of the microbiota, including Chao 1 (A), observed species (B), phylogenetic diversity (PD) whole tree (C), and Shannon index (D). Especially, observed species were decreased by 71.94% (556 to 156) and 76.08% (556 to 133) by administration with the antibiotic cocktail for 1 or 3 weeks (*P* < 0.05), respectively.

### The Effect of Antibiotic Cocktail on Microbiota Composition at the Phylum Level

**FIGURE 3:**
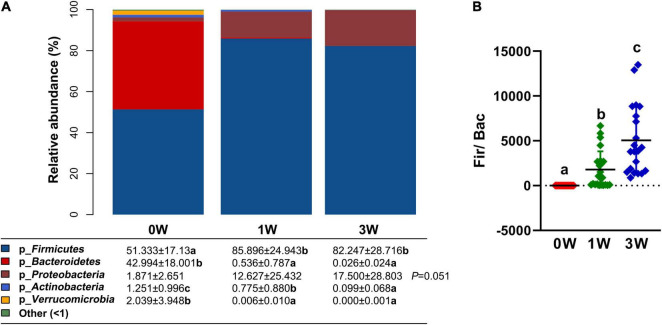
The relative abundance of fecal microbiota at the phylum level. **(A)** The relative abundance of microbes. **(B)** The ratio of Firmicutes to Bacteroidetes. Data represented as mean ± SD (*n* = 24). Bars with different letters differ significantly (*P* < 0.05). p_ represents microbial phylum.

To make an insight into the alteration of gut microbiota after treatment with an antibiotic cocktail, the relative abundance of microbes at the phylum level in different periods was analyzed. The relative abundance of 3 kinds of phyla including *Bacteroidetes*, *Actinobacteria*, and *Verrucomicrobia* was decreased significantly by the antibiotic cocktail (*P* < 0.05). In detail, Bacteroidetes was decreased by 98.75 and 99.94%, *Actinobacteria* was decreased by 38.05 and 92.09%, while *Verrucomicrobia* was decreased by 99.71 and 100%, respectively, after treatment with the cocktail for 1 or 3 weeks (Figure 3A). *Firmicutes* dominated the gut microbiota, and the ratio of *Firmicutes* to *Bacteroidetes* was increased by the antibiotic cocktail in a dose-dependent manner (Figure 3B).

### The Effect of Antibiotic Cocktail on Microbiota Composition at the Genus Level

**FIGURE 4:**
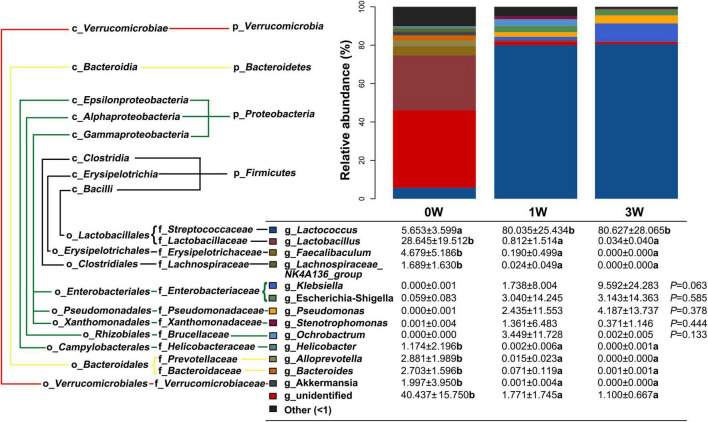
The relative abundance of fecal microbiota at the genus level. Data represented as mean ± SD (*n* = 24). Bars with different letters differ significantly (*P* < 0.05). g_ represents microbial genus.

Further analysis of the relative abundance of microbes at the genus level revealed that *Lactococcus*, a genus belonging to the phylum of *Firmicutes*, was increased significantly after being treated with the antibiotic cocktail for 1 or 3 weeks (*P* < 0.05), and became the dominant genus (80.63%) in the microbial community (Figure 4). On the other hand, *Lactobacillus*, *Faecalibaculum*, and *Lachnospiraceae_ NK4A136_group* belonging to the phylum of *Firmicutes*, *Helicobacter* belonging to the phylum of *Proteobacteria*, *Alloprevotella*, and *Bacteroides* belonging to the phylum of *Bacteroidetes*, and *Akkermansia* belonging to the phylum of *Verrucomicrobia* were decreased significantly by the antibiotic cocktail (*P* < 0.05).

### Efficiency of Fecal Microbiota Transplantation on Antibiotics-Pretreated Mice

**FIGURE 5:**
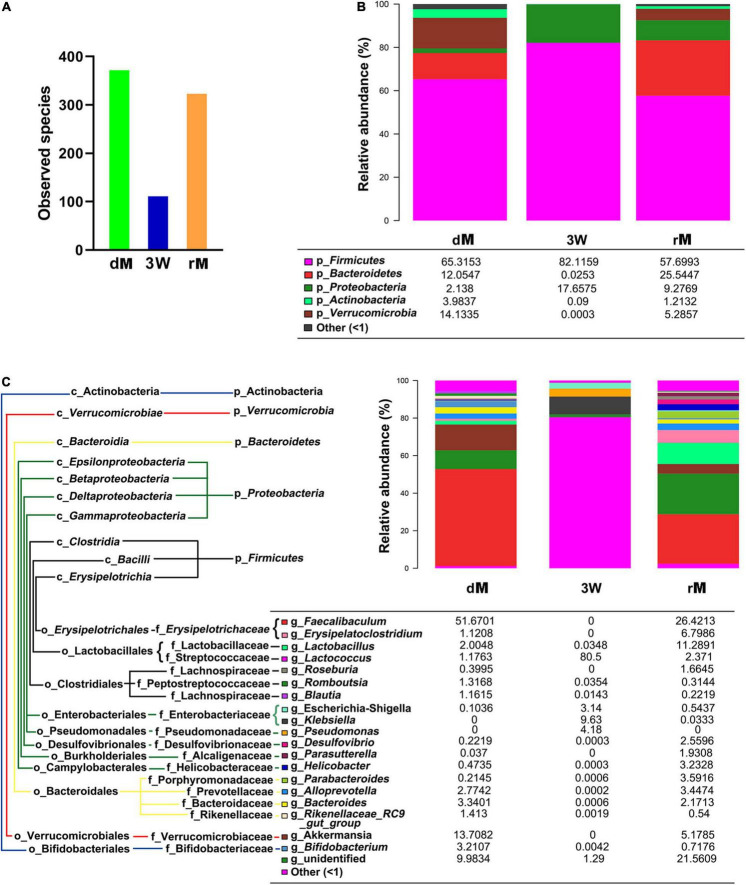
The diversity and relative abundance of fecal microbiota after FMT. **(A)** Observed species of fecal microbiota among donor mice (dM), mice treated with the antibiotic cocktail for 3 weeks (3W), and receptor mice (rM). **(B)** Effect of FMT on the relative abundance of fecal microbiota at the phylum level. **(C)** Effect of FMT on the relative abundance of fecal microbiota at the genus level. dM: donor mice fed with normal diet; FMT, fecal microbiota transplantation; rM, receptor mice with FMT from dM; p_ represents microbial phylum, g_ represents microbial genus.

To verify whether the pretreatment of the antibiotic cocktail is effective, a further FMT experiment was conducted. As shown in Figure 5, the observed species in receptor mice (rM) were elevated obviously as compared with antibiotics-pretreated mice at the 3rd week (3 W). Meanwhile, the number of observed species in rM was similar to that in donor mice (dM) (Figure 5A). Further analysis of the composition of relative gut microbiota at phylum or genus level revealed that FMT reversed relative gut microbiota composition, with more abundant taxon as well as similar relative gut microbiota composition to dM (Figures 5B,C).

## Discussion

Driven by its amenable property, the antibiotic cocktail serves as the conventionally applicable method to deplete indigenous microbiota to make receptors obtain a susceptible status. In the present study, an antibiotic cocktail containing vancomycin (0.5 g/L), ampicillin (1 g/L), neomycin (1 g/L), and metronidazole (1 g/L) was used in the mouse model for FMT. Vancomycin and ampicillin mainly suppress gram-positive bacteria by inhibiting the synthesis of cell walls (18). Neomycin can inhibit the synthesis of protein in aerobes (19), and metronidazole can inhibit the synthesis of DNA in anaerobes (20). In this study, the observed species of indigenous microbiota were depleted by 71.94% (from 556 to 156) and 76.08% (from 556 to 133), mainly including *Bacteroidetes*, *Actinobacteria*, and *Verrucomicrobia*, after being treated with the antibiotic cocktail for 1 and 3 weeks, respectively, but recovered by the subsequent FMT with fecal microbiota from normal mice. Moreover, treatment with the cocktail increased significantly the ratio of *Firmicutes* to *Bacteroidetes*, which was similar to the results of an antibiotic cocktail containing ampicillin (1 g/L) and neomycin (0.5 g/L) for 2 weeks (21). Unexpectedly, *Lactococcus*, a genus belonging to the phylum of *Firmicutes*, dominated the gut microbiota of mice at the genus level after treating with the antibiotic cocktail either for 1 or 3 weeks. The relative abundance of *Lactobacillus* and *Akkermansia* were also decreased significantly by administrating with the antibiotic cocktail for 1 or 3 weeks, which was in accordance with previous studies (14, 22). However, the relative abundance of Proteobacteria had large individual differences in this study, while another study reported that the alpha subclass of Proteobacteria can be decreased significantly by the antibiotic cocktail (23).

Antibiotics directly target antibiotic-sensitive bacterial species and then affect other microbes owing to the dysbiosis (24). Recent studies have suggested that *Lactococcus* (such as *Lactococcus lactis subsp. lactis*) may act as the predator bacterial to exert their antibacterial property *via* the generation of bacteriocins (25), and an antibiotic cocktail of ciprofloxacin and metronidazole in drinking water *ad libitum* to mice for 2 weeks can enhance the relative abundance of *Lactococcus lactis* (26). Potentially, the antibiotic cocktail containing vancomycin, ampicillin, neomycin, and metronidazole may cause a relative increase in predators bacterial such as *Lactococcus* (25) and pro-inflammatory bacteria such as *Escherichia-Shigella* and *Klebsiella* (27), but decrease the relative abundance of anti-inflammatory bacteria such as *Akkermansia* (28) and autoimmunity-driven bacteria such as *Lactobacillus* (29), which further affect the capability of microbiota in controlling intestinal inflammation and immunity (30), with a susceptible micro-environment (31) ready for FMT.

Collectively, the antibiotic cocktail effectively depleted indigenous microbiota, and is available for the pretreatment of FMT. Nevertheless, considering the dominant genus *Lactococcus* after treatment with the antibiotic cocktail, extra data in different situations are needed to ensure disease-specific attributes of donors.

## Conclusion

Treatment with an antibiotic cocktail containing vancomycin (0.5 g/L), ampicillin (1 g/L), neomycin (1 g/L), and metronidazole (1 g/L) in drinking water for 3 weeks eliminated microbes belonging to *Bacteroidetes*, *Actinobacteria*, and *Verrucomicrobia* in the gut microbiota of mice, and those microbes can be recovered by FMT.

## Data Availability Statement

The datasets presented in this study can be found in online repositories. The names of the repository/repositories and accession number(s) can be found in the article/Supplementary Material.

## Ethics Statement

The animal study was reviewed and approved by the Hunan Agricultural University Institutional Animal Care and Use Committee.

## Author Contributions

JT, JG, FL, BL, ZL, and JY: experimental execution. JT and SW: writing – original draft preparation. SW and JH: writing – review and editing and supervision. All authors contributed to the article and approved the submitted version.

## Conflict of Interest

The authors declare that the research was conducted in the absence of any commercial or financial relationships that could be construed as a potential conflict of interest.

## Publisher’s Note

All claims expressed in this article are solely those of the authors and do not necessarily represent those of their affiliated organizations, or those of the publisher, the editors and the reviewers. Any product that may be evaluated in this article, or claim that may be made by its manufacturer, is not guaranteed or endorsed by the publisher.

## Supplementary Material

Supplementary Table 1 Diet composition of mouse feed.
