# Supplementary material for: Evaluation of an Antibiotic Cocktail for Fecal Microbiota Transplantation in Mouse
Source: Front Nutr. 2022 Jun 3;9:918098. doi: 10.3389/fnut.2022.918098 (PMC9204140; doi:10.3389/fnut.2022.918098)
Supplement: Supplementary Table 1 — Diet composition of mouse feed. [file Data_Sheet_1.pdf]

Supplemental Table 1 Diet composition of Mouse Feed

D12450J		
	gm%	kcal%
Protein	19.2	20
Carbohydrate	67.3	70
Fat	4.3	10
Total		100
**kcal/gm**	**3.85**	
Ingredient	gm	kcal
Casein, 30 Mesh	200	800
L-Cystine	3	12
Corn Starch	506.2	2024.8
Maltodextrin 10	125	500
Sucrose	68.8	275.2
Cellulose, BW200	50	0
Soybean Oil	25	225
Lard*	20	180
Mineral Mix S10026	10	0
DiCalcium Phosphate	13	0
Calcium Carbonate	5.5	0
Potassium Citrate, 1 H2O	16.5	0
Vitamin Mix V10001	10	40
Choline Bitartrate	2	0
FD&C Yellow Dye #5	0.04	0
FD&C Blue Dye #1	0.01	0
Total	1055.1	4057
